# Enhanced fluorescent imaging of proteins in live yeast cells using fluorescently labeled scFv

**DOI:** 10.1016/j.xpro.2023.102299

**Published:** 2023-06-02

**Authors:** Ioannis Tsirkas, Tomer Zur, Daniel Dovrat, Zohar Paleiov, Lior Ravkaie, Amir Aharoni

**Affiliations:** 1 Department of Life Sciences and the National Institute for Biotechnology in the Negev, Ben-Gurion University of the Negev, Be’er Sheva 84105, Israel

**Keywords:** Cell Biology, Single Cell, Microscopy, Molecular/Chemical Probes

## Abstract

Fluorescent labeling of proteins is a widespread approach for the microscopic examination of protein function, expression, and localization in the cell. Here, we present a protocol for the labeling of hemagglutinin (HA)-tagged protein of interest (POI) with the single-chain antibody (scFv) 2E2 fused to different fluorescent proteins (FPs) in *Saccharomyces cerevisiae*. We describe steps for expressing 2E2-FP, and HA tagging and labeling of POI. We detail *in vivo* fluorescent imaging of proteins at different cellular compartments and with diverse expression levels.

For complete details on the use and execution of this protocol, please refer to Tsirkas et al. (2022).^1^

## Highlights

• Enhanced fluorescent labeling of HA-tagged proteins in live yeast cells with 2E2-FP
• Collection of plasmids containing 2E2 scFv fused with green, red, and blue FPs and HALOtag
• Step-to-step guide for HA-labeling of POI and 2E2-FP expression
• Identification and quantification of ring-shaped structures and foci/puncta formation

## Abstract (editor highlights)

Publisher’s note: Undertaking any experimental protocol requires adherence to local institutional guidelines for laboratory safety and ethics.

## Graphical abstract

**Figure:**
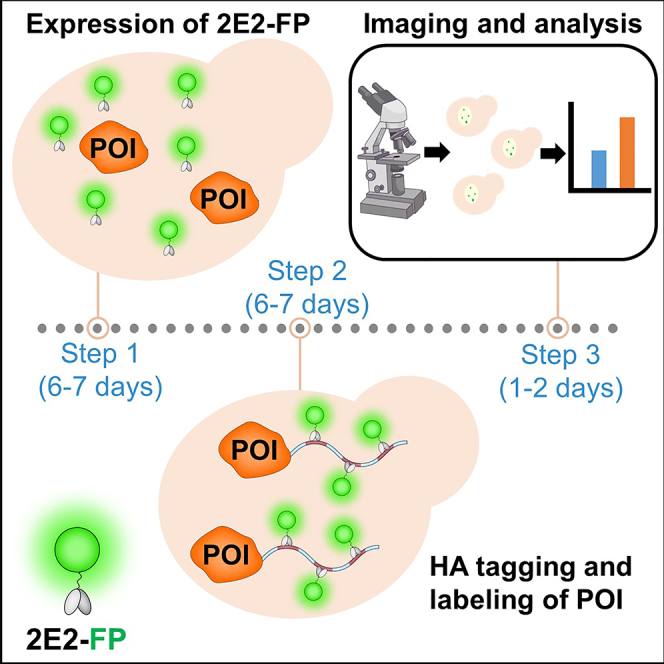

## Before you begin

This protocol describes the fluorescent labeling of a hemagglutinin (HA)-tagged POI by 2E2-FP in live yeast cells. Specifically, the protocol describes a stepwise approach for the efficient genomic integration of 2E2-FP expression cassettes, C-terminal HA-tagging of a POI with 3HA or 6HA repeats, and live cell microscopy imaging of the fluorescently labeled POI in yeast. Prior to lab experiments, this protocol describes the different versions of 2E2-FP available for labeling an HA tagged POI, allowing the reader to choose the best version suited for their needs. These 2E2-FP versions include 2E2-FP expressed from two different promoters, different FPs including Envy (green), mKate2 (red), and Electra1 (blue), or HALOtag, the presence or absence of a nuclear localization sequence (NLS) and different selection marker cassettes. The second step describes how to choose and design primers for HA tagging of POI.

### Choosing the 2E2-FP version for labeling your HA tagged POI in live cells

**Timing: 10–20 min**

**Figure 1:**
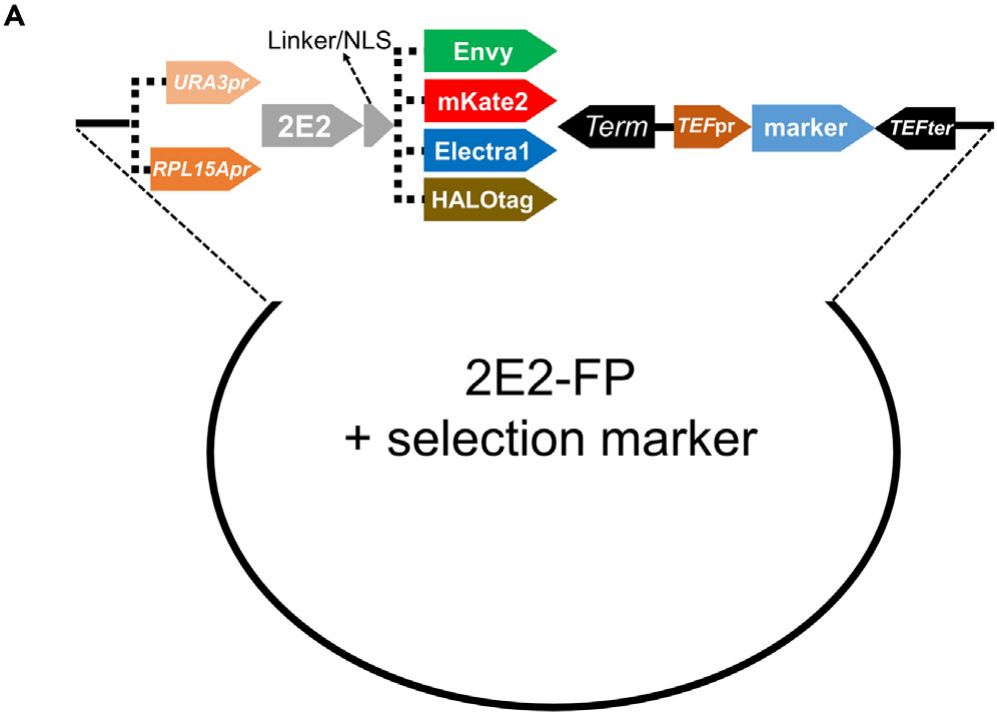
Detailed plasmid scheme depicting the available 2E2-FP expression constructs The constructs are expressed by either a weak truncated *URA3* or a strong *RPL15A* promoter (shades of orange) (See Table 3 for promoter sequences). 2E2 is fused to a green (Envy), red (YOmKate2) or blue (Electra1) FP, or HALOtag (brown) through a GS-rich linker, with or without a NLS (grey). Expression is terminated by either *ADH1* or *CYC1* terminators (black). Selection gene markers *kanMX*, *hphMX*, *C.a. URA3*, or *S.p. HIS5*) (light blue) are expressed under *TEF* promoter (orange) and terminator (black). Envy and mKate2 plasmids (pTZ1 and pTZ2 respectively) can be integrated into the *ADE1* locus (*SrfI* restriction digestion). HALOtag construct under the expression of *URA3* promoter (pTZ4A) can be integrated in the *URA3* locus (*PacI* restriction digestion). HALOtag constructs under the expression of *RPL15A* promoter can be integrated into the *PDR5* locus (*SrfI* restriction digestion) (See Table 1 and text for details).

We have constructed a collection of plasmids containing 2E2 scFv^1^^,^^2^ fused to three different FPs, that are characterized by high brightness and/or are well suited for yeast imaging: a yeast-optimized (YO) version of GFP (Envy, Ex: 488, Em: 510),^3^ a YO version of mKate2 (Ex: 588, Em: 633),^4^ and a blue fluorescent protein (Electra1, Ex: 402, Em: 454)^5^ (Figure 1 and Table 1). Additionally, we fused 2E2 with HALOtag,^6^ for the labeling of proteins with a variety of HALOtag-compatible dyes, for example *silicon rhodamine* (SiR, Ex: 652, Em: 674). The 2E2-FP constructs are expressed either from a 175 bp short version of the *URA3* promoter for obtaining low expression or from a 623 bp *RPL15A* promoter for obtaining a higher expression level^7^ (Figure 1 and Table 1). Transcription of the 2E2-FP is terminated by either *CYC1* or *ADH1* terminators. The *kanMX* and *hphMX* antibiotic resistance cassettes or *URA* and *HIS* selection genes are integrated upstream or downstream of the 2E2-FP constructs (Figure 1 and Table 1).

1. Choose the optimal version of 2E2-FP-selection marker for labeling your HA tagged POI.

***Note:*** Selection should be made based on the desired FP for labeling your POI, the expression level of the POI and selection marker suitability (Table 1). *RPL15A* promoter constructs are recommended for most use cases, especially when the expression level of the POI is relatively high. The *URA3* promoter is preferable when the expression of the POI is low. Additionally, for efficient protein labeling with 2E2-HALOtag, the *PDR5* gene needs to be deleted in order to achieve efficient retention of the HALOtag-compatible small-molecule dyes within the yeast cells.^8^ W1588 MATa *S. cerevisiae* strains expressing the different 2E2-FP are available upon request for the labeling of HA tagged POI. [See troubleshooting Problem 1]

***Note:*** Plasmids **pTZ1** and **pTZ2** are *ADE1*-integrative. Plasmid **pTZ3** can be integrated by amplification of the construct-selection marker and subsequent yeast transformation. Plasmid **pTZ4A** is *URA3-*integrative*.* Plasmids **pTZ4B and pTZ4C** are *PDR5*-integrative, in order to achieve expression of the 2E2-HALOtag construct and deletion of *PDR5* gene in one step, allowing successful labeling with HALOtag-compatible dyes.^8^ Nevertheless, all constructs can be amplified and integrated in the desired locus (*URA3*, *HO*, etc.). See also Figure 1.

**Table 1:** Plasmids for the expression of 2E2 fused to Envy, YOmKate2, Electra1, and HALOtag

Plasmids	Promoter	Localization signal	Selection marker	Integration (Restriction Site)
**pTZ1A** 2E2-Envy	*URA3*	NLS	*kanMX*	*ADE1 (SrfI)*
**pTZ1B** 2E2-Envy	*URA3*	NLS	*hphMX*	*ADE1 (SrfI)*
**pTZ1C** 2E2-Envy	*URA3*	–	*kanMX*	*ADE1 (SrfI)*
**pTZ1D** 2E2–Envy	*URA3*	–	*hphMX*	*ADE1 (SrfI)*
**pTZ1E** 2E2–Envy	*RPL15A*	–	*hphMX*	*ADE1 (SrfI)*
**pTZ2A** 2E2–mKate2	*URA3*	NLS	*kanMX*	*ADE1 (SrfI)*
**pTZ2B** 2E2–mKate2	*URA3*	NLS	*hphMX*	*ADE1 (SrfI)*
**pTZ2C** 2E2–mKate2	*URA3*	–	*kanMX*	*ADE1 (SrfI)*
**pTZ2D** 2E2–mKate2	*URA3*	–	*hphMX*	*ADE1 (SrfI)*
**pTZ2E** 2E2–mKate2	*RPL15A*	–	*hphMX*	*ADE1 (SrfI)*
**pTZ3** 2E2–Electra1	*RPL15A*	–	*kanMX*	–
**pTZ4A** 2E2–HALOtag	*URA3*	NLS	*C.a. URA3*	*URA3 (PacI)*
**pTZ4B** 2E2–HALOtag	*RPL15A*	NLS	*S.p. HIS5*	*PDR5 (SrfI)*
**pTZ4C** 2E2–HALOtag	*RPL15A*	–	*S.p. HIS5*	*PDR5 (SrfI)*

### Choosing and designing primers for HA tagging of your POI

**Timing: 15–30 min**

**Figure 2:**
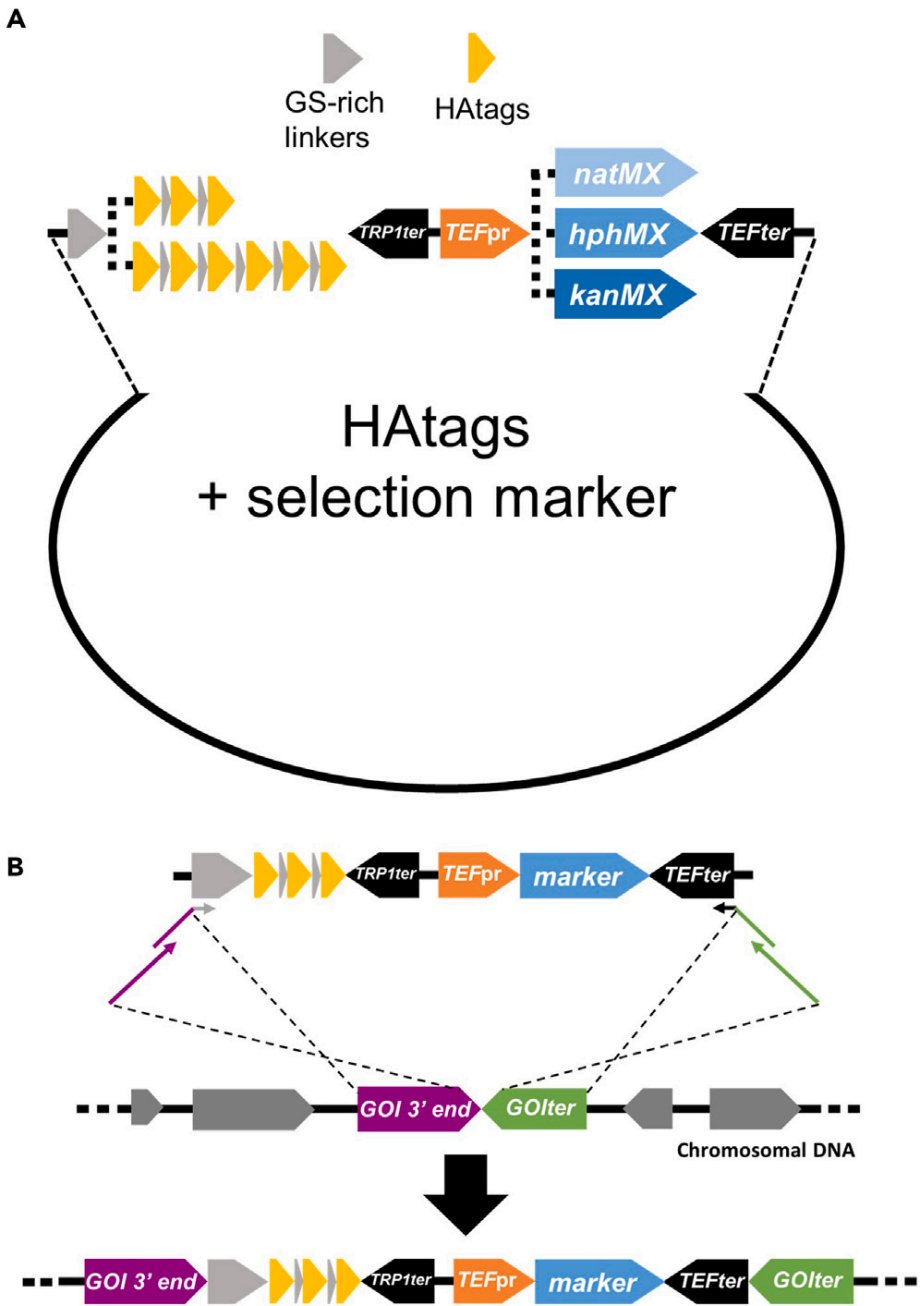
HAtags-selection marker plasmid design and strategy for tagging the 3′ end of a gene of interest (GOI) (A) Detailed plasmid scheme showing the available plasmids for HA-tagging of GOI (See also Tables 2 and 3). Readers can choose to integrate 3HA or 6HA repeats (yellow) and among three antibiotic selection markers, *natMX*, *hphMX*, *kanMX* (shades of blue). HA-tags are separated from each other and from the GOI with GS-rich linkers (grey). HA-labeled protein expression is terminated by *TRP1* terminator (black). Expression of antibiotic selection markers is controlled by *TEF* promoter (orange) and terminator (black). (B) Amplification strategy of HA-cassettes for labeling a GOI at 3′ end at its native genomic locus. Primers with partial homology to the 12AA GS-rich linker (grey) and *TEF* terminator (black) are used to amplify the cassettes and introduce homology to the GOI 3′ end (excluding stop codon) and GOI terminator (purple and green, respectively) (See Tables 2 and 3). Additional primers can be designed to introduce longer homology with the region of interest (See also text).

We have constructed plasmids containing three and six repeats of the HA epitope, 3HA and 6HA, respectively, downstream of a 12 amino-acid (AA) GS-rich linker, for the C-terminus HA tagging of a POI (Figure 2A, Tables 2 and 3). Non-repetitive 8 AA GS-rich linkers separate the HA epitopes to increase the efficiency of 2E2-FP labeling and ensure facile amplification and integration of the HA cassette into the yeast genome (Figure 2, Tables 2 and 3). Different codons were used for the same AAs to avoid repetitiveness of the HA epitopes and linker sequences. A short *TRP1* terminator is placed downstream of the HA cassette for efficient transcription termination of the HA tagged gene (Figure 2 and Table 3). These plasmids also contain an antibiotic resistance cassette (*natMX*, *hphMX*, or *kanMX*),^9^ under control of *TEF* promoter and terminator, for the facile selection of yeast transformants containing the HA tagged gene of interest (GOI) (Figure 2, Tables 2 and 3).

***Note:*** If the GOI is already tagged with HA repeats, this step can be ignored.

**CRITICAL:** For strains containing HA tagged POI, ensure that the HA repeats are separated from the POI and from each other with flexible linkers of sufficient length (8 AAs GS-rich linkers are used in this protocol) (Table 3).

2. Choose the suitable version of 3HA- or 6HA-antibiotic marker for tagging your POI, based on the preferred selection markers and properties of POI (Figure 2A and Table 2).

***Note:*** Tagging POI with 6HA, compared to 3HA, can lead to increased 2E2-FP recruitment to the tagged POI. This may result in reduced 2E2-FP background signal and improved signal-to-noise ratio. Additionally, it can improve the detection of the HA tagged POI by western blot (WB) analysis. However, in cases where the POI is sensitive to C-terminus tagging, the shorter 3HA is preferable.

3. Design and order primers for 3HA or 6HA tagging at the 3′-end of your POI encoding gene.

***Note:*** For the amplification of the 3HA and 6HA cassettes, the forward (Frw) and reverse (Rev) primers should anneal to the 5′ of the linker sequence and the 3′ of the *TEF* terminator (pAG25-HA and pTZ-HA plasmids) or promoter (pAG32-HA plasmids), respectively, appending ≥ 50 bp of homology to the GOI and terminator region (Figure 2B). The 5′ of the PCR product should be homologous to the end of the GOI, excluding the stop codon, while the 3′ of the PCR product should be homologous to the GOI terminator. To improve the integration efficiency, longer primers can be used to generate higher homology to the GOI and terminator (primer length of up to 100 bp can be used). Alternatively, additional Frw and Rev primers can be designed for further amplification of the initial PCR product described above, to increase the homology and integration efficiency (Figure 2B).

**CRITICAL:** Terminator sequences in yeast tend to be AT-rich and thus, primers for cassette amplification may generate secondary structures which can hinder the PCR reaction. In these cases, primer design guidelines should be considered carefully before ordering.

**Table 2:** Plasmids containing the 3HA or 6HA-antibiotic resistance cassette

Plasmids	Antibiotic marker
pAG25-3HA	*natMX*
pAG25-6HA	
pAG32-3HA	*hphMX*
pAG32-6HA	
pTZ-3HA	*kanMX*
pTZ-6HA	

See also Figure 2.

**Table 3:** Sequences of linkers, HA epitopes, *URA3* or *RPL15A* promoters*,* and *TRP1* terminator

Sequence type	Amino acid sequence (N → C)	Nucleotide sequence (5′ → 3′)
GOI-HA-linker	SGSASGGGGSGG	AGTGGGTCTGCTAGCGGTGGAGGTGGGAGTGGAGGC
HA sequence	YPYDVPDYA	TATCCTTACGACGTCCCCGATTACGCA
		TATCCGTATGACGTACCCGATTATGCT
		TACCCATACGACGTTCCCGACTACGCT
		TATCCGTATGATGTGCCAGACTATGCA
		TATCCATATGATGTTCCTGATTACGCC
		TACCCCTATGATGTACCAGACTATGCG
Linker between HA sequences	GGSGGSGG	GGTGGCAGTGGGGGGTCAGGCGGA
	GGGSGGSG	GGAGGTGGTTCAGGTGGCTCAGGA
	GGGSGGSG	GGTGGCGGCTCTGGTGGCTCAGGT
	GGSGGSGG	GGAGGTTCCGGCGGTAGCGGAGGC
	GSGGGGSG	GGCTCAGGAGGGGGCGGAAGCGGT
*URA3* promoter	–	-175 GTTTCTTTGA….AAGATAAATC -1
*RPL15A* promoter	–	-623 GTATTCAAGA…TAAATCAGCA -1
*TRP1* terminator	–	GTTATTACTGAGTAGTATTTATTTAAGTATTGTTTGTGCACTTGCCT

## Key resources table

**Table:** 

REAGENT or RESOURCE	SOURCE	IDENTIFIER
**Chemicals, peptides, and recombinant proteins**		

Difco™ Yeast Nitrogen Base w/o AAs (YNB) (powder)	Becton, Dickinson and Company	Cat# 291940
Bacto™ agar (powder)	Becton, Dickinson and Company	Cat# 214010
Bacto™ yeast extract (powder)	Becton, Dickinson and Company	Cat# 212750
Bacto™ peptone (powder)	Becton, Dickinson and Company	Cat# 211677
YPD broth (powder)	Formedium	Cat# CCM0210
D-(+)-Glucose, anhydrous, 99% (powder)	Alfa Aesar	Cat# A16828
Drop-out Mix Complete (powder)	US Biological Life Sciences	Cat# D9515
G418 Disulfate salt (G418) (powder)	Formedium	Cat# G4185
Hygromycin B (HYG) (powder)	Formedium	Cat# HYG5000
Nourseothricin sulfate (ClonNAT) (powder)	GoldBio	Cat# N-500-3
Water (H_2_O)	Sigma-Aldrich	Cat# 7732-18-5
*SrfI* + rCutsmart	New England Biolabs	Cat# R0629S
*PacI* + rCutsmart	New England Biolabs	Cat# R0547S
Agarose (powder)	Life Gene	Cat# LAG0701
SYBR Safe DNA Gel Stain	Invitrogen	Cat# S33102
DNA Ladder 1 kb/ 100 bp	New England Biolabs	Cat# N3231/2 L
Purple Loading Dye	New England Biolabs	Cat# B7024A
TAE buffer 50×	Biolab	Cat# 002050232300
Sodium hydroxide 98% (NaOH) (powder)	Acros Organics	Cat# 134070010
Herring Sperm DNA (liquid)	Promega	Cat# D1816
Lithium acetate dihydrate (LiAc) (powder)	Sigma-Aldrich	Cat# L6883
Poly(Ethylene glycol) 3,350 (PEG) (powder)	Sigma-Aldrich	Cat# P4338
Glycerol anhydrous	Biolab	Cat# 000712050100
Tris-HCl (powder)	Thermo Fisher Scientific	Cat# BP153-1
Sodium chloride (NaCl) (powder)	Biolab	Cat# 001903059100
Manganese chloride (MnCl_2_) (powder)	Strem Chemicals	Cat# 93-2527
Calcium chloride dihydrate (CaCl_2_·2H_2_0) (powder)	Thermo Fisher Scientific	Cat# 10035-04-8
Concanavalin A (ConA) (powder)	Sigma-Aldrich	Cat# L7647
Silicon-rhodamine HALO dye	Tsirkas et al.^1^	N/A

**Critical commercial assays**		

KOD Hot Start DNA Polymerase	Millipore	Cat# 71086-3
KAPA polymerase and buffers	Roche	Cat# 07958846001
ALLIn™ RED Taq Mastermix 2×	HighQu	Cat# PCM0201c1
DreaMTaq Green PCR Master Mix 2×	Thermo Fisher Scientific	Cat# K1081

**Deposited Data**		

Microscopy and UV agarose gel images	This manuscript	https://doi.org/10.5281/zenodo.7398277
HALOtag labeling microscopy images	This manuscript	https://doi.org/10.5281/zenodo.7756480

**Experimental models: Organisms/strains**		

*Saccharomyces cerevisiae* W1588	Tsirkas et al.^1^ and this manuscript	N/A

**Oligonucleotides**		

DNA oligos for PCR amplification and plasmid construction	IDT and Sigma-Aldrich	N/A

**Recombinant DNA**		

Plasmids for 2E2-FP integration and expression	Tsirkas et al.^1^ and this manuscript	N/A
Plasmids for -3HA or -6HA protein labeling	Tsirkas et al.^1^ and this manuscript	N/A

**Software and algorithms**		

Matlab (version R2019b)	MathWorks	https://www.mathworks.com/products.html?s_tid=gn_ps
Python (versions 2.7 and 3.8)	Python	https://www.python.org
PyCharm (2020.2)	JetBrains	https://www.jetbrains.com/pycharm/
ZEN (version 3.0 blue edition)	Zeiss	https://www.micro-shop.zeiss.com/en/us/softwarefinder/#select-soft-ware
ImageJ	ImageJ	https://imagej.net/downloads
TokyoGhoulRe (foci/background identification and quantification)	Tsirkas et al.^1^	https://doi.org/10.5281/zenodo.7214818
EldenRing (nuclear membrane identification and quantification)	Tsirkas et al.^1^	https://doi.org/10.5281/zenodo.7214832

**Other**		

μ-Slide 8 Well Uncoated	Ibidi	Cat# 80821
Petri dishes	Greiner Bio-one	Cat# 633 102
PCR strip tubes	Axygen	Cat# PCR-0208-C
PCR tubes	Axygen	Cat# PCR-02-A
Weighing paper	Bar Naor Ltd	Cat# BN70081L
Laboratory film	Parafilm	Cat# PM-996
Eclipse™ Pipet Tips 10–20 μL	Labcon	Cat# 1036-260-000-9
Eclipse™ Pipet Tips 100-200-250 μL	Labcon	Cat# 1093-260-000-9
Eclipse™ Pipet Tips 1000–1250 μL	Labcon	Cat# 1045-260-000-9
Discovery Comfort Pipette 0.5–10 μL, 2–20 μL, 20–200 μL, 100–1000 μL	HTL	Cat# 7901
5 mL pipettes	Costar	Cat# 4487
10 mL pipettes	Sorfa	Cat# 314100
25 mL pipettes	Sorfa	Cat# 315100
1.5 mL Eppendorf tubes	FL Medical	Cat# 23053
1.5 mL black Eppendorf tubes	N/A	Cat# HS4323K
2.0 mL Cryovial tubes	Thermo Fisher Scientific	Cat# 1154P75
50 mL falcon tubes	N/A	Cat# 227270
Quadloops PS	N/A	Cat# 8150032001
Filters 0.22 μm (200-5000-1000 mL)	N/A	N/A
Filter 0.4 μm for syringes	N/A	N/A
Cell Spreaders	N/A	N/A
Plastic Volumetric Cylinders (100-500-1000 mL)	N/A	N/A
Glass Bottles (250-500-1000 mL)	N/A	N/A
Microscope Stand Axio Observer 7	Zeiss	Cat# 431007-9904-000
Scanning Stage 130 × 100 STEP (D)	Zeiss	Cat# 432029-9904-000
Stage Controller XY STEP SMC 2009	Zeiss	Cat# 432929-9011-000
Solid-State Light Source Colibri 7	Zeiss	Cat# 423052-9741-000
Photometrics sCMOS Prime BSI imaging camera	Teledyne Photometrics	Cat# 01-PRIME-BSI-R-M-16-C
Nanodrop 2000 Spectrophotometer	Thermo Fisher Scientific	N/A
Tabletop Microcentrifuge MicroCL 17R	Thermo Fisher Scientific	N/A
Tabletop Centrifuge5810R for 50 mL tubes	Lumitron	N/A
Weighing scale	Presica	N/A
Analytical scale	Sartorius	N/A
C1000 Touch Thermal Cycler	Bio-Rad	N/A
Vortex Genie 2	Scientific Industries	N/A
SevenEasy™ pH Meter	Mettler Toledo	N/A
Biophotometer	Eppendorf	N/A
Microwave oven	N/A	N/A

## Materials and equipment

### YPD medium

Dissolve 25 g of YPD Broth in 500 mL ddH_2_0 and autoclave. Let cool down and store at 4°C for up to 6 months.

**Table:** YPD Agar Plates

Reagent	Final concentration	Amount
YPD Broth	N/A	25 g
Bacto™ Agar	2%	10 g
ddH_2_O	N/A	up to 500 mL
**Total**	**N/A**	**500 mL**

Dissolve 25 g of YPD Broth and 10 g of agar in 500 mL ddH_2_0 and autoclave. When the medium becomes lukewarm, add the desired antibiotic (G418 = 400 μg/mL, HYG = 200 μg/mL, ClonNAT = 100 μg/mL), mix, and transfer 25 mL to 100 mm petri dishes. Let cool down and store at 4°C for up to 6 months.

### 50% PEG 3350

Dissolve 40 g PEG 3350 in ddH_2_O up to final volume 80 mL and autoclave. Store at 25°C for up to 12 months.

### LiAc 1M

Dissolve 6.12 g LiAc in 60 mL ddH_2_O and autoclave. Store at 25°C for up to 12 months.

**Table:** Synthetic Complete (SC) Medium

Reagent	Final concentration	Amount
YNB w/o AAs	N/A	6.7 g
DO Mix Complete	N/A	2 g
D-Glucose	4%	40 g
ddH_2_O	N/A	950 mL
**Total**	**N/A**	**1000 mL**

Dissolve 6.7 g YNB w/o AAs, 2 g DO Mix Complete, 40 g D-Glucose in 950 mL of ddH_2_O. Mix by stirring and filter through a 1 L 0.22 μm vacuum filter. Store at 4°C for up to 6 months.

***Note:*** SC Medium 2% Glucose can also be used instead of 4%.

### Boiled Herring sperm DNA

Aliquot 50 μL Herring sperm DNA in 1.5 mL Eppendorf tubes and boil at 100°C for 5–10 min. Store at −20°C for up to 12 months.

### 50% glycerol

Mix equal amount of glycerol with ddH_2_O and autoclave. Store at 25°C for up to 12 months.

### NaOH 1 M

Dissolve 2 g NaOH in 50 mL ddH_2_O on ice. For a final solution of 20 mM NaOH, dissolve NaOH 1 M in ddH_2_O 50 times (50×). Store at 25°C for up to 12 months.

**CRITICAL:** Sodium hydroxide (NaOH) is an extremely caustic chemical that can cause severe burns by all exposure routes. Proper protective equipment (e.g., lab coats, gloves, goggles, etc.) should be used when handling NaOH pellets and solutions – consult the SDS.

**Table:** ConA buffer pH = 6.8

Reagent	Final concentration	Amount
Tris-HCl	20 mM	315 mg
NaCl	200 mM	1169 mg
CaCl_2_·2H_2_O	1 mM	14.702 mg
MnCl_2_	1 mM	12.584 mg
ddH_2_O	N/A	up to 100 mL
**Total**	**N/A**	**100 mL**

Dissolve solid compounds in 70 mL ddH_2_O and adjust the pH to 6.8 with NaOH 1 M. Then complete with ddH_2_O up to 100 mL. ConA Buffer can be stored at 4°C for up to 6 months.

### ConA solution 2 mg/mL

Dissolve 20 mg of ConA in 10 mL ConA Buffer. Mix until almost complete dissolution of white precipitates and then filter through a 0.4 μm filter with a syringe. Aliquot 800–850 μL in 1.5 mL Eppendorf tubes and store at −20°C for up to 12 months. To coat the surface of the 8-well Ibidi slides, add 200 μL 2 mg/mL ConA in Buffer in each well and incubate for 1 h up to 12 h. Pipette out the ConA solution from the wells and let dry at 25°C before using. ConA on microscopy slides/wells can be stable at 25°C for several weeks [See troubleshooting Problem 2].

## Step-by-step method details

This protocol describes the integration of the 2E2-FP expression cassette (steps 1–3), the 3HA or 6HA tagging of a POI in yeast (steps 4–6), and the fluorescent imaging of the generated yeast strains under the microscope (step 7). If a yeast strain expressing the desired 2E2-FP (Table 1) is available, steps 1–3 can be ignored. In case a strain with 3HA or 6HA tagged POI is available, steps 4–6 can be ignored. Furthermore, steps 4–6 can precede steps 1–3.

### Integration and expression of 2E2-FP

**Timing: 6–7 days**

Steps 1–3 include the integration of a 2E2-FP gene into the yeast genome followed by imaging of the expressed 2E2-FP protein in yeast cells.

1. Restriction digestion of the desired 2E2-FP plasmid(s) (See Figure 1 and Table 1).
  a. Digest 1–2 μg of the desired 2E2-FP plasmid with 0.5 μL of the appropriate restriction enzyme, according to manufacturer’s guidelines.
  b. Verify the successful digestion of the 2E2-FP plasmid.
    i. Load 100 ng of the digested plasmid and a sample of 1 kb DNA ladder onto a 1% agarose gel, stained with SYBR SAFE DNA Gel Stain, inside a gel-running device filled with TAE buffer.
    ii. Run the gel for 30 min, 120 V and visualize under blue light or UV lamp.

**Figure 3:**
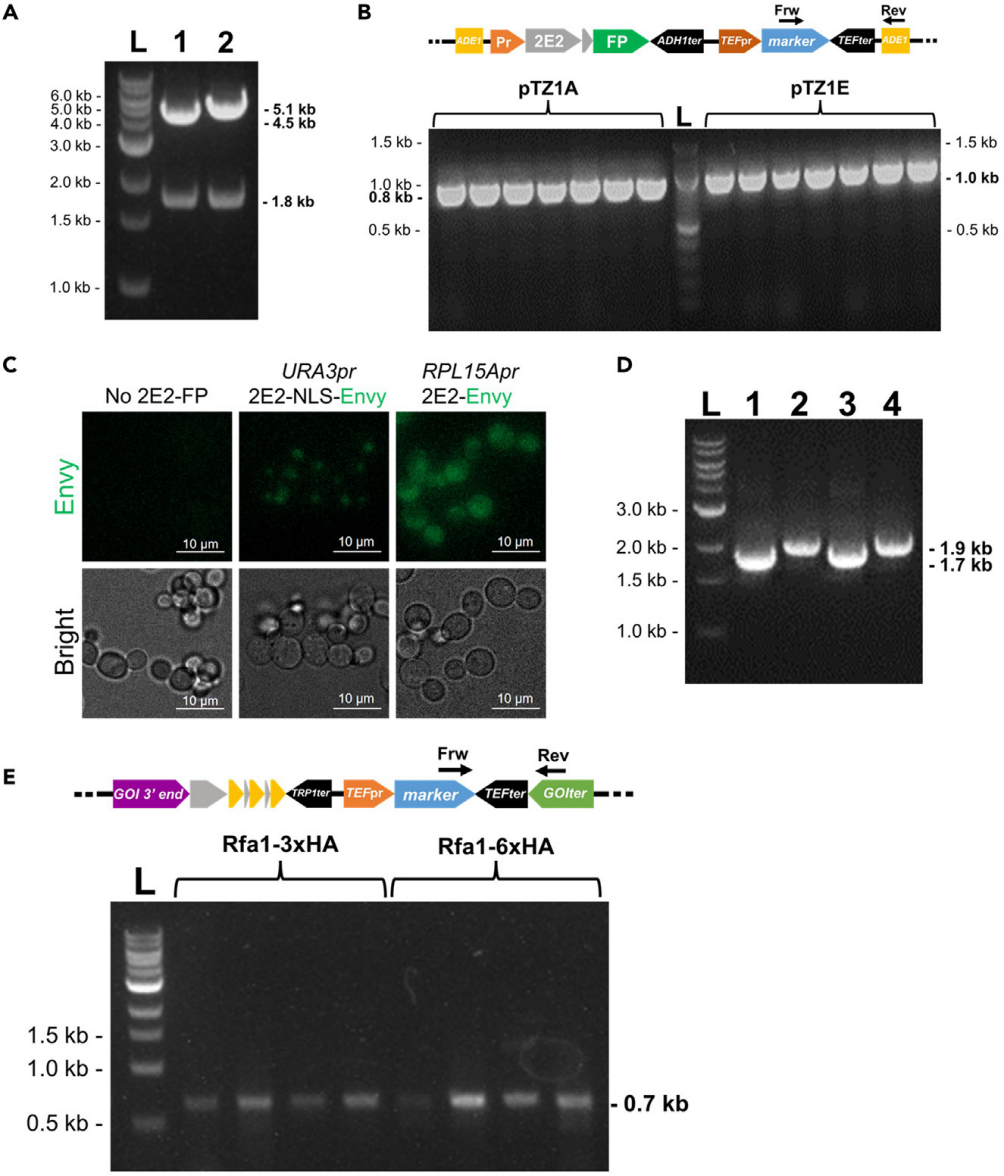
Generation and validation of strains expressing 2E2-FP and HA-tagged GOI (A) Agarose gel UV photo showing the 1 kb DNA Ladder (L), and 100 ng of *SrfI*-restricted pTZ1A (1) or pTZ1E (2) plasmids (Table 1). The 4.5 kb and 5.1 kb DNA bands correspond to the *URA3pr*-*2E2-NLS-Envy-kanMX* (1) or *RPL15Apr-2E2-Envy-hphMX* (2) cassettes respectively. The lower 1.8 kb DNA bands correspond to plasmid backbone. (B and C) Validation of 2E2-Envy integration in the yeast genome. (B) Top: Scheme of the 2E2-Envy-marker construct inside the yeast genome (*ADE1* locus), showing the annealing regions of the Frw and Rev primers used in the validation of successful integration (See Table 5). Expected PCR product sizes are 0.8 kb (*KanMX* Rev and *Ade1Locus Frw* primers) and 1 kb (*HphMX* Frw and *Ade1Locus* Rev) for pTZ1A and pTZ1E, respectively. Bottom: UV photo of agarose gel showing the validation PCR for colonies tested for the integration of the *SrfI*-restricted pTZ1A (7 colonies, left) or pTZ1E (7 colonies, right) (Figure 3A). All tested colonies were positive, as indicated by the strong PCR bands of the correct size. (C) Representative microscopy images of unlabeled yeast cells, or cells expressing 2E2-Envy constructs. Cells containing *URA3pr*-*2E2-NLS-Envy* (pTZ1A) present weak nuclear signal, while those with *RPL15Apr-2E2-Envy* (pTZ1E) show a non-localized stronger signal. (D) UV photo of agarose gel depicting the successful amplification of 3HA (1 and 3) and 6HA (2 and 4) with primers homologous to GOIs (Rfa1 and Nup159 genes for 1–2 and 3–4 respectively). See also Figure 2B. (E) As in (B), but for the validation of 3HA and 6HA integration at the C-terminus of the GOI (tagging of Rfa1 is shown as an example). Top: Scheme of the HA-tagged GOI inside the yeast genome. Expected PCR product size is 0.7 kb (Frw primer: *NatMX* Frw) (See Table 5). Bottom: All tested colonies (Rfa1-3HA-*natMX* – 4 colonies, left and Rfa1-6HA-*natMX* – 4 colonies, right), were positive as indicated by the PCR bands of the correct size. In (B) and (E), 10 μL PCR products were loaded in the gel.

***Note:*** A successful restriction of the pTZ1, pTZ2, and pTZ4 plasmids leads to two distinct bands: a longer band containing the 2E2-FP cassette with flanking homologous sequences to the integrative locus (4.3 kb–5.5 kb) and a shorter band containing the plasmid backbone (1.8 kb) (Figure 3A). In case non-integrative plasmid is selected, the 2E2-FP-selection marker cassette needs to be amplified and integrated in the desired genomic locus.

**Pause Point:** Restriction digested plasmids can be stored at −20°C until further use.

2. Yeast transformation with the restriction digested 2E2-FP cassette for genome integration.
  a. Inoculate yeast cells for transformation, from an agar plate or frozen glycerol stock, into 5 mL prewarmed YPD medium or an appropriate selective medium in a 50 mL falcon tube. Grow at 30°C in a shaker incubator.
    ***Note:*** If not stated otherwise, all incubation steps take place at 30°C in a shaker incubator.
  b. Next day, measure O.D._600_ of the yeast culture and dilute cells to O.D._600_ of 0.1 in 12 mL prewarmed YPD medium. Incubate for approximately 3 h or until O.D._600_ = 0.35–0.50 is reached.
  c. Wash step: Centrifuge yeast culture at 3000 × *g* for 5 min. Discard supernatant and wash twice with 10–15 mL ddH_2_O.
  d. Resuspend washed cell pellet with 100 μL ddH_2_O.
  e. Prepare a transformation reaction in an Eppendorf tube, according to Table 4.

**Table 4:** Yeast transformation reaction components

Reagent	Volume
PEG 3350 50%	480 μL
LiAc 1 M	72 μL
Boiled Herring sperm DNA	10 μL
Digested plasmid	1–2 μg (25–50 μL)
Yeast cells	≈50–100 μL
  f. Mix by inverting, briefly vortex the transformation tube, and spin down briefly to collect any solution from the walls of the tube.
  g. Incubate the transformation reaction at 42°C in a shaker for 40–60 min.
  h. Centrifuge cells at 12000 × *g* for 1 min. Discard supernatant.
  i. Recovery step: Resuspend cells in 1–2 mL prewarmed YPD medium. Transfer resuspended cells to a 50 mL falcon tube and incubate for 2 h.
    **CRITICAL:** Recovery step can be avoided when 2E2-HALOtag constructs (pTZ4) are integrated, as non-antibiotic selection is used in these cases.
  j. Centrifuge cells at 3000 × *g* for 5 min.
  k. Discard supernatant and resuspend with 100 μL of sterile ddH_2_O.
  l. Transfer the desired fraction of transformed cells on selective agar plates and spread cells using a spreader.
    ***Note:*** A few tens of positive colonies are expected to grow following transformation. However, transformation efficiency may vary depending on the target strain and DNA amount, thus, it is suggested plating multiple dilutions of the transformed cells to achieve the optimal outcome (e.g., 1/2, 1/5, 1/10, 1/20). [See troubleshooting Problem 3]
  m. Incubate the agar plates with the transformed cells at 30°C for 2–3 days.

3. Selection, validation, and storage of positive colonies.
  Two to three days following yeast transformation, colonies should be visible on the selective agar plate.
  a. Pick 4–10 single yeast colonies and spread the cells on a selective agar plate with quadloops or pipette tips. Incubate for approximately 20 h at 30°C.
    **Pause point:** Transformation and colony plates can be stored at 4°C, wrapped with parafilm, for 1–2 weeks, but it is recommended to continue immediately with the next steps.
  b. Validate successful transformants with PCR.
    ***Note:*** ALLIn™ RED Taq Mastermix or DreamTaq Green PCR Master Mix, as well as other polymerases, can be used in the PCR validation of successfully transformed yeast colonies. Table 5 includes a list of primers which recognize unique sequences on the antibiotic cassettes or *ADE1* locus which can be used for 2E2-FP integration and HA-labeling validation (Figure 3B).
    i. Prepare a Master Mix in an 1.5 mL Eppendorf tube according to manufacturer’s guidelines.
    ii. Split into aliquots of 19.5 μL in PCR tubes according to the number of colonies tested.
      ***Note:*** Keep all PCR reagents and mixtures on ice prior to initiating the PCR reaction.
    iii. Inoculate a small amount of yeast colonies into separate Eppendorf tubes containing 50 μL NaOH 20 mM.
    iv. Vortex the tubes with yeast cells and heat at 100°C for 5 min to lyse the yeast cells.
    v. Vortex briefly and centrifuge tubes at 20000 × *g* for 1 min.
    vi. Transfer and mix 0.5 μL of each supernatant into the tubes with the PCR reaction.
    vii. Spin down briefly, place inside a thermocycler, and initiate the PCR reaction protocol, according to manufacturer’s guidelines.
    viii. Verify the successful amplification as described in step 1b by loading 10 μL of the PCR reactions in agarose gel (Figure 3B) [See troubleshooting Problem 4].

**Table 5:** Primers sequence for validation of 2E2-FP and HA cassettes integration in the yeast genome

Name	Sequence (5′ → 3′)	Description
***KanMX* Rev**	CAGCATCCATGTTGGAATTTAATCGCGG	Validation of *kanMX* integration
***NatMX* Frw**	GCGCTCTACATGAGCATGCCCTGC	Validation of *natMX* integration
***HphMX* Frw**	GCTCTCGATGAGCTGATGCTTTGG	Validation of *hphMX* integration
***HphMX* Rev**	CATCAGGTCGGAGACGCTGTC	
***HphMX* Rev2**	ATCGGCGAGTACTTCTACACAGCC	
***TEF*term Frw**	GCTGGTCGCTATACTGCTGTCG	Validation of *TEF*term integration
***TEF*term Rev**	CGAATCGACAGCAGTATAGCGACCAGC	
***Ade1Locus* Frw**	CAGGCCTTCCACTTTTGAATTACTGC	Validation of 2E2-FP integration in *ADE1* locus
***Ade1Locus* Frw2**	AGCGAGCCAGGGAAGTAATTAGCG	
***Ade1Locus* Rev**	AACTCCTTAAGTACTACCACAGCCAG	
***Ade1Locus* Rev2**	GTCTGACTCTTGCGAGAGATGAGG
  c. Image positive colonies and long-term storage.
    i. Inoculate the yeast colonies into 3 mL prewarmed SC medium in 50 mL falcon tubes. Vortex briefly and grow at 30°C in a shaker.
      ***Note:*** SC medium should always be used for microscope imaging instead of YPD, as YPD medium results in high background fluorescence.
    ii. Next day, measure the O.D._600_ of the yeast cultures and dilute cells to O.D._600_ = 0.1–0.15 in 2 mL prewarmed SC medium in 50 mL falcon tubes. Grow for 2–3 h at 30°C in a shaker to O.D._600_ = 0.3–0.5.
    iii. Transfer 300–500 μL of yeast cultures into separate wells of uncoated 8-well Ibidi slides.
    iv. Image yeast cultures with a wide-field or confocal fluorescence microscope for examining the expression level of 2E2-FP with the appropriate light source and beam splitter-filter set (Figure 3C).
      **CRITICAL:** Along with the 2E2-FP yeast colonies, a WT strain, lacking any fluorescent marker, should be imaged simultaneously for comparison, especially in the case of 2E2-FP expressed from the weak *URA3* promoter (Figure 3C). In case a 2E2-FP version with NLS is used, only the cell nucleus should be fluorescently labeled (Figure 3C).
    v. Inoculate one or two colonies in 5 mL prewarmed YPD medium, supplemented with the appropriate antibiotic reagent(s). Grow yeast cultures at 30°C in a shaker.
    vi. Next day, mix yeast cultures with 50% glycerol in 1:1 ratio in Eppendorf or Cryovial tubes.
    vii. Store glycerol stocks at −80°C.
      **Pause Point:** Yeast strains can be stored long-term as glycerol stocks at −80°C and used for inoculation into prewarmed medium for future use.
      ***Note:***Table 5 includes primers used during the validation of yeast strains expressing 2E2-FP and HA-labeled genes. The primers names specify the entity they bind. Primers numbered with “2” indicate alternative primers which can be used for the same gene/locus.

### Tagging a POI with 3HA or 6HA in cells expressing 2E2-FP

**Timing: 6–7 days**

Steps 4–6 include the amplification and integration of the 3HA or 6HA cassette for tagging a POI in yeast cells expressing a 2E2-FP.

4. Amplification of the HA cassette (Table 2) with a high-fidelity polymerase.
  ***Note:*** KOD HOT Start and KAPA Hifi DNA Polymerases are two common polymerases utilized for such amplifications, but, in principle, any high-fidelity polymerase can be used. Reagent amounts and conditions suggested by the Taq polymerase manufacturer should be applied.
  a. Prepare a Master Mix containing four to eight 25 μL PCR reactions in an Eppendorf tube.
    i. Pipette 25 μL into PCR tubes or strip tubes (100–200 μL total PCR product).
  b. Use a 2-step PCR cycling protocol (Table 6).
    ***Note:*** Keep all PCR reagents and mixture on ice prior to initiating the PCR reaction.
    ***Note:*** The annealing temperature in 1st step of the PCR cycling conditions should be selected given the annealing sequence of the Frw and Rev primers to the HA cassette, as determined by the user (Figure 2B).

**Table 6:** PCR cycling conditions for KOD HOT Start DNA Polymerase for HA cassette amplification

Steps	Temperature	Time	Cycles
Polymerase activation	95°C	2 min	1

**1**^**st**^**step (Primers annealing to the HA cassette and initial amplification)**			

Denaturation	95°C	20 s	5
Annealing	(According to primers designed by the user) °C	20 s	
Extension	70°C	45 s	

**2**^**nd**^**step (Final amplification)**			

Denaturation	95°C	20 s	30
Annealing	(Average of full-length Frw and Rev primers Tm) °C	20 s	
Extension	70°C	45 s	
Final extension	70°C	3 min	1
Hold	4°C	ꝏ	

See also Figure 2.
  c. Verify the successful amplification of the HA cassette as in step 1b. The expected size should be approximately 1.7 kb and 1.9 kb for 3HA and 6HA respectively (Figure 3D).
    ***Optional:*** To improve genomic integration of the HA cassette, extension primers can be utilized for further amplification of the PCR product leading to increased homology with the gene of interest, as described in step 3 of primer design.
    **Pause Point:** PCR products can be stored at −20°C until further use.

5. Transformation of the 3HA or 6HA cassettes in yeast strains expressing 2E2-FP as described in step 2.

***Note:*** Use 100–200 μL of PCR product from step 4 for the yeast transformation in place of the “digested plasmid” listed (Table 4).

6. Selection, validation, and storage of successfully HA-transformed yeast colonies as in step 3.

***Note:*** Here, the Frw and Rev primers for validation of HA cassette recognize unique sequences on the antibiotic cassette(s) or downstream of the GOI terminator, respectively (Figure 3E and Table 5).

**Pause Point:** Transformants and colony plates can be stored at 4°C, wrapped with parafilm, for 1–2 weeks, but it is recommended to continue immediately with the next steps. Yeast strains can be long-term stored at −80°C as glycerol stocks and inoculated into prewarmed medium for imaging.

### Live cell fluorescent imaging of HA tagged POI labeled with 2E2-FP

**Timing: 1–2 days**

This step describes the microscopic examination of HA-tagged POIs labeled by the 2E2-FP.

7. Fluorescent imaging of 2E2-FP labeled HA-tagged proteins in live yeast cells.
  a. Inoculate the yeast strains from an agar plate or glycerol stock into 5 mL prewarmed SC medium in a 50 mL falcon tube. Vortex briefly and grow at 30°C in a shaker.
  b. Next day, measure the O.D._600_ of the yeast cultures. Dilute cells to O.D._600_ = 0.1–0.15 in 2 mL prewarmed SC medium in 50 mL falcon tubes. Grow for 2–3 h in a shaker at 30°C to O.D._600_ = 0.3–0.5.
  c. Briefly vortex cell cultures and transfer 300–500 μL of yeast cultures into separate wells of ConA-coated 8-well Ibidi slides for immobilization.
  d. Incubate at 25°C for 10–15 min according to the desired cell confluence on the slide.
    ***Note:*** If O.D._600_ ≈ 0.4, then transfer 350 μL of cell cultures into wells and incubate for 10 min for a final well confluence ≈ 50%–70%.
  e. Washing steps: Remove the medium from the wells and gently pipette fresh 500 μL prewarmed SC medium into the wells and discard. Repeat 1–2 times.
  f. Add 350 μL prewarmed SC medium into the wells.
  g. Image the immobilized yeast cells with a wide-field or confocal microscope inside a temperature-controlled enclosure.
    i. Turn on the fluorescent microscope and set temperature to 30°C to sustain normal yeast physiology at least 2–3 h prior to the microscopy experiment.
    ii. Open the imaging software.
    iii. Choose the objective lens (e.g., 40×, 63×, 100× etc.) according to the magnification preference which best fits your experiment.
    iv. For optimal resolution use 1 × 1 binning in the camera settings, if applicable.
    v. Use the appropriate light source - beam splitter - filter set to excite and detect the selected fluorescent protein (Excitation with 390 nm, 488 nm, 590 nm, and 640 nm for Electra1, Envy, mKate2, and Silicon Rhodamine respectively). Use bright-field or DIC to visualize yeast cell morphology.
    vi. Adjust illumination intensity and exposure time according to the fluorescent intensity of the labeled protein, so that a strong and clear signal can be obtained, for further quantification and analysis.
    vii. To detect fluorescent signal throughout the whole cell body, acquire multiple Z-slices (e.g., 12 Z-slices with 800 nm steps).
    viii. In time-lapse experiments, excite cells periodically in the preferred intervals for the desired time period.
    ix. Save microscopy experiment output (photos or video files) and proceed with image analysis.
      ***Note:*** Fluorescent imaging protocol and conditions vary according to the user’s preferences and experiment type.
      **CRITICAL:** We suggest, when imaging yeast strains expressing an HA-tagged protein with 2E2-FP for first time, to simultaneously image yeast cells expressing the POI directly fused with a FP, either at C- or N-terminal ends. Alternatively or simultaneously, readers can consult previously published results or online databases (e.g., SGD, LoQAtE^10^) with information about the localization of fluorescently labeled yeast proteins. These controls and preparations can serve as *bona fide* comparison and validation of the 2E2-FP labeling efficiency.

## Expected outcomes

**Figure 4:**
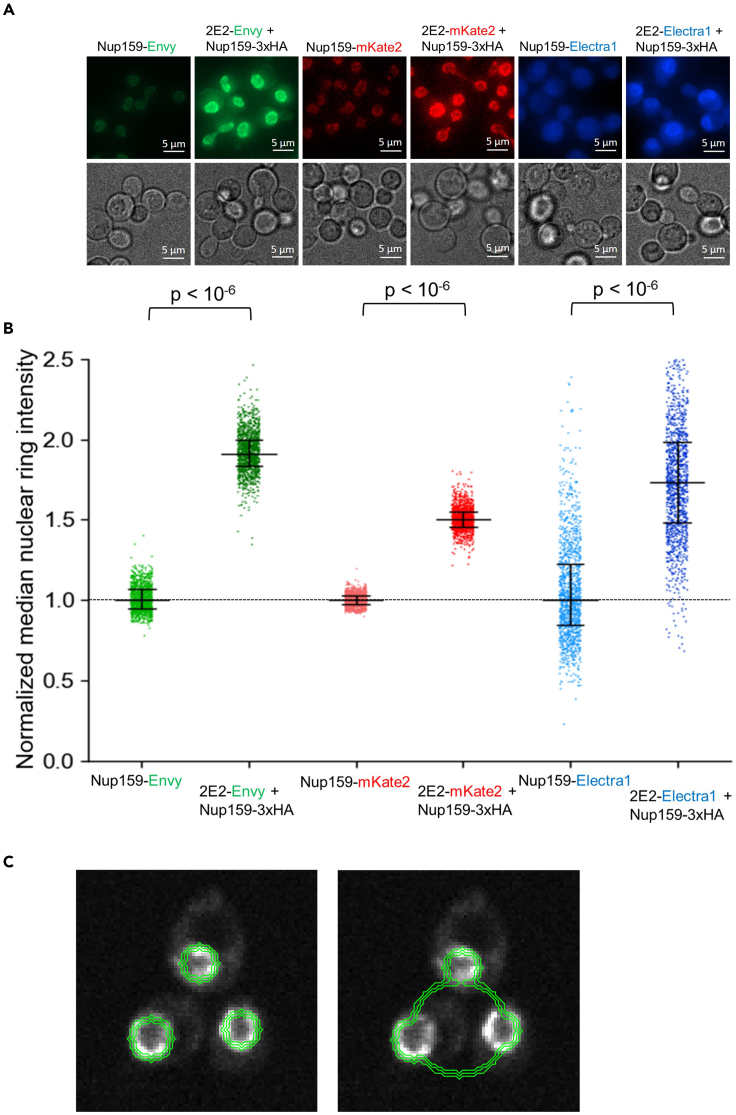
Enhanced imaging of Nup159 by 3HA labeling and expression of 2E2 fused to three different fluorescent proteins (A) Representative fluorescent microscopy photos (single Z slices) of cells expressing Nup159 fused to Envy, mKate2, and Electra1 or labeled Nup159-3HA cells expressing 2E2-Envy, 2E2-mKate2, and 2E2-Electra1 from the strong *RPL15A* promoter. In all cases, Nup159-3HA labeled strains expressing the respective 2E2-FP proteins present stronger nucleoporin fluorescent signal. (B) Quantification and comparison of nuclear ring intensities of 1116, 1231, and 1368 cells with Nup159 fused to Envy, mKate2, or Electra1, respectively and 1326, 1227, and 1313 cells expressing Nup159-3HA and 2E2-Envy, 2E2-mKate2, or 2E2-Electra1, respectively. A black dashed line highlights the median normalized intensity of the Nup159 direct-FP labeled yeast strains. Normalized median values and 25%–75% quartiles are shown. For statistical analysis, nuclear ring intensities were compared with Monte Carlo resampling with 1,000,000 iterations. In Electra1-labeled strains, cytoplasmic fluorescent signal was removed using ImageJ to facilitate nuclear ring identification. (C) Examples of fluorescently-labeled Nup159 nuclear rings identification using our custom-made MATLAB script ‘’EldenRing’’. Left: Correct identification of fluorescently labeled nuclear membrane limits in single cells. Green lines passing over the labeled nuclear membranes highlight the region included in the quantification. Right: False identification of nuclear membranes limits in single cells, as a result of very low and/or high ‘’radius’’ parameter or extreme sensitivity values selection. See text for more details.

**Figure 5:**
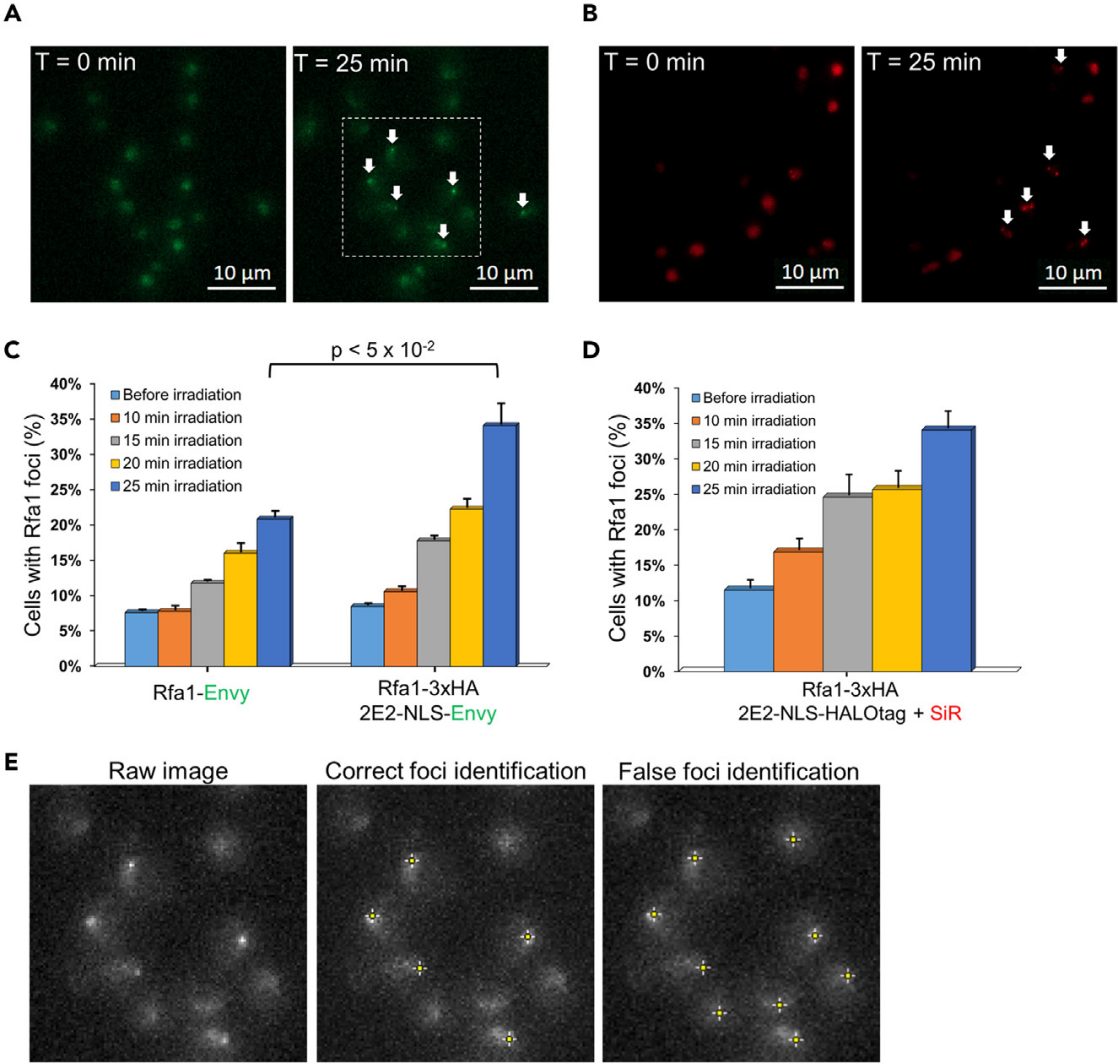
Optimized detection of RPA foci with 3HA-labeled Rfa1 and 2E2-NLS-FP variants (A and B) Representative fluorescent microscopy images of cells expressing Rfa1-3HA and (A) 2E2-NLS-Envy or (B) 2E2-NLS-HALOtag with the addition of the far-red dye SiR-HALO, from the weak *URA3* promoter, before (T = 0 min) and 25 min after irradiation with 390 nm light at 1-min intervals. The appearance of multiple Rfa1 foci is visible (white arrows). A white, dashed box surrounds the region shown in E). (C and D) Detection of cells with Rfa1 foci in Rfa1-3HA cells labeled with 2E2-NLS-Envy (C) or 2E2-NLS-HALOtag + SiR-HALO (D) expressed under the weak *URA3* promoter after 10, 15, 20, and 25 min of irradiation. For calculating the percentage of cells with Rfa1 foci, the number of cells with foci was divided with the total number of cells on the imaging field for the presented timepoints. A combination of automated and manual foci identification was performed. Results shown are averages of triplicates, with at least 170 cells in each replicate. Statistical analysis was performed with One-Way ANOVA. Error bars are standard error of the mean (SEM). Different illumination conditions were applied to 2E2-NLS-Envy and 2E2-NLS-HALOtag + SiR-HALO Rfa1-3HA labeled cells during DNA damage induction. (E) Examples of correct (middle) and false (right) foci identification inside the white dashed region of (A) (left) using our custom-made MATLAB-ImageJ combined script “TokyoGhoulRe”. Yellow-white crosses mark the identified foci (local maxima). Right panel shows false-positive foci identification as a result of small noise parameter value selection during identification (see text and troubleshooting Problem 5 for more details).

The expected outcomes from this protocol are the enhanced fluorescent imaging of HA tagged POI labeled with 2E2-FP in live yeast cells that provide several important advantages over direct fusion of FP to POI. First, labeling of HA tagged POIs allows a simple and effective mean for fluorescent signal amplification due to the ability of multiple 2E2-FPs to bind to the 3HA or 6HA labeled POIs. We demonstrate such signal amplification for Nup159-3HA labeled with *RPL15A*pr-2E2-FP, relative to the direct fusion of Nup159 to FP (Figure 4). Second, the background fluorescence level in the yeast cell is independent of the expression level of the POI and depends only on the 2E2-FP expression. Thus, for the sensitive detection of fluorescent foci and enhanced signal-to-noise ratio, a weak promoter for 2E2-FP can be used. We demonstrate the increased detection of DNA damage foci in Rfa1-3HA labeled with 2E2-NLS-Envy expressed from the weak *URA3* promoter, relative to Rfa1-Envy fusion (Figure 5). Third, the small size of the HA epitopes does not impede the function of the labeled protein, in contrast to the significantly larger FPs. Again, enhanced labeling of Rfa1-3HA in comparison to direct Envy-labeling highlights this advantage (Figure 5). Fourth, the availability of four versions of 2E2-FP including the 2E2-Envy, 2E2-mKate2, 2E2-Electra1, and 2E2-HALOtag allows high flexibility of fluorescent labeling of HA tagged POI. This can be particularly important for the simultaneous imaging of more than one fluorescently labeled POI in yeast cells. We demonstrate such flexibility for the labeling of Nup159-3HA with 2E2-Envy, 2E2-mKate2, and 2E2-Electra1, as well as Rfa1-3HA labeling with 2E2-NLS-Envy and 2E2-NLS-HALOtag with Silicon Rhodamine-HALO dye (SiR-HALO^11^) (Figures 4 and 5). Finally, the availability of 2E2-FP expressed from the weak *URA3* promoter and the stronger *RPL15A* promoter allows the labeling of a variety of yeast proteins exhibiting different expression levels. In a previous publication, we have demonstrated the ability of the 2E2-FP to label HA tagged Rad52, Nup157, Sec7, Pex3, and Tom70 located in the nucleus, nuclear membrane, Golgi, peroxisomes, and mitochondria, respectively.^1^

## Quantification and statistical analysis

Quantification and statistical analysis of the fluorescent images from experiments with HA-tagged POI labeled with 2E2-FP largely depend on the experiment type, the subcellular localization of the POI, and the nature of the biological observations or outputs. Here, we present two distinct examples of fluorescent quantification using custom-made MATLAB codes. The first example allows the quantification of nuclear membrane fluorescence intensity in cells expressing Nup159-3HA labeled with 2E2-FP expressed from the stronger *RPL15A* promoter. The second example describes the identification of RPA foci in cells expressing Rfa1-3HA with 2E2-NLS-Envy or 2E2-NLS-HALOtag and SiR-HALO following DNA damage induction by periodic near-UV irradiation. However, these types of analysis can be applied for the identification and quantification of other proteins visualized as ring-shaped structures or distinct foci/puncta under the fluorescent microscope.

Steps in quantification and statistical analysis:

1. Generate maximum intensity Z projections.
2. Export microscopy images as single-channel .tif files. Give a simple and easily distinguishable name to each file (e.g., image1, image2, etc). Transfer all generated files to be analyzed in the same folder.

**CRITICAL:** Avoid any processing of the images, such as deconvolution, which may alter the original pixel intensities, and, thus, affect subsequent quantification.

3. Run the custom-made MATLAB codes for the identification and quantification of either ring-shaped structures (EldenRing) or foci/puncta (TokyoGhoulRe) as described below.
  a. Membrane perimeter identification and signal quantification (Figure 4).
    i. Activate Matlab software and open the ‘’**EldenRing**’’ script.
    ii. Within the “EldenRing” script, the following parameters should be changed according to desired preferences:
    iii. Enter the path for the directory where the generated .tif files reside.
      Folder ='**C:\path'**;
    iv. Enter the dimensions of the .tif files (in pixels) as parameter **W** in the following line:
      img = img(10: (**W**-10), 10: (**W**-10));
    v. Modify the following parameters as needed, in order to optimize the identification of ring-shaped structures in the microscopy images (Figure 4C). The parameters define the minimum and maximum diameter of the ring to be identified, and the sensitivity for ring detection. These parameters should be determined empirically depending on the magnification, cell/nucleus size, and signal-to-noise ratio [See troubleshooting Problem 5]
      [centers,radii] = imfindcircles(img,[**6 40**], ‘Sensitivity’, **0.85**);
    vi. Run ‘’EldenRing’’ in Matlab command window. The output will be an excel file with name ‘’**ringsIntensities.xlsx**’’. Each column contains the file name, average and median nuclear membrane intensities, and the nucleus diameter for every cell.
  b. Determination and quantification of fluorescent foci (Figure 5).
    i. Install the MIJ package in Matlab, which enables running ImageJ and Fiji through Matlab, and follow the online guidelines:
    ii. Activate Matlab software and open the ‘’**TokyoGhoulRe**’’ script.
    iii. Enter the path for the directory where the generated .tif files reside.
      Folder = ‘**C:\path**’;
    iv. Enter a value in the noise parameter (**X**) according to the signal-to-noise ratio of the foci.
      MIJ.run(‘Find Maxima’…‘, 'noise=**X** output=List exclude');
      ***Note:*** The noise value should be determined before the user runs the code for first time. Open one or more of the generated .tif files in **ImageJ** and choose **Process**->**Find_Maxima**. Try different values for the **Prominence**/**Noise_Tolerance** field, select **Point_selection** in the **Output_type,** and activate ☑ **Preview_point_selection**. By gradually increasing the value of the **Noise_Tolerance**, less foci will be marked on the open .tif file in the ImageJ window. Choose the value which best resembles selected foci, excluding background (Figure 5E). Use this **Prominence**/**Noise_Tolerance** value on the noise field of the “TokyoGhoulRe” script. [See troubleshooting Problem 5]
      **CRITICAL:** While small modifications on the noise value have small effect on the number of identified foci, the same value must be used for all your samples/images analysis, as this parameter defines the threshold between background and local maxima.
    v. Enter the number of pixels **Y** and the radius **Z** around the local maxima for foci quantification. These parameters depend on the type of the labeled POI (e.g., foci/puncta size, protein accumulation/localization) and the imaging specifications (e.g., magnification, fluorescence intensity, etc.) and they need to be determined empirically by carefully determining foci size and morphology from multiple cells.
      pixelNumber = **Y**;
      radius = **Z**;
    vi. Enter the dimensions of the .tif files (in pixels) as parameter **W** in the following line:
      (size(img,1)== **W**);
      pixels = **W**-Z;
    vii. Run ‘’TokyoGhoulRe’’ in Matlab command window. If all parameters have been inserted correctly, the output will be an excel file with name ‘’**fociIntensities.xlsx**’’. Each column contains a file name and the foci intensities for this file.

4. Compare data output for each experimental condition/strain from replicates using the appropriate statistical test (student’s t-test, ANOVA, etc).
5. Present results with the desired graph format (histogram, box, swarm, dot, strip, violin plots, etc.) (Figures 4 and 5).

## Limitations

The labeling efficiency of HA-tagged proteins with 2E2-FP is dependent on the expression ratio of POI-HA and 2E2-FP. Consequently, if the expression of the POI is significantly higher than that of the 2E2-FP, there will not be enough 2E2-FP molecules to bind on the 3HA or 6HA tagged POIs. Analogously, if the expression of the POI is much lower than the 2E2-FP, the unbound 2E2-FP molecules may increase the fluorescent background. This can be solved by assessing the labeling efficiency with both *URA3* and *RPL15A*-expressed 2E2-FP and choosing the optimal promoter. Our previous experiments indicate that proteins that are sensitive to C-terminal labeling such as Rfa1 and/or proteins that form large oligomeric structures such as Rad52 are optimally labeled with 2E2-FP expressed from the weak *URA3* promoter.^1^ Furthermore, although this has not been observed in any of the labeled proteins we tested, we cannot exclude the possibility that the HA-tagging of a protein and the expression of the 2E2-FP may result in synthetic lethality in yeast cells. An additional limitation is the labeling of proteins residing in cellular compartments inaccessible by 2E2-FP. Nevertheless, this can be overcome with the addition of the appropriate localization signal sequence to the 2E2 constructs. Furthermore, since the fluorescent signal of the labeled proteins is determined by the association of multiple 2E2-FP species on the HAtags, the current methodology is not suitable for molecular abundance estimation experiments. Finally, proteins that exhibit diffused localization, or are extremely sensitive to C-terminal tagging, although still possible, are not ultimately compatible to the HA and 2E2-FP labeling approach.

## Troubleshooting

### Problem 1

The fluorescent signal of the HA-labeled protein is very weak, non-specific or the background fluorescence is very strong.

### Potential solution

The version of the 2E2-FP which is used to label the HA-tagged protein is of utmost importance to achieve optimal fluorescent labeling in live cells. This can be dependent on the expression level of both the POI and the 2E2-FP, as well as their localization (e.g., nuclear, cytoplasmic, mitochondrial etc). We have demonstrated that in highly expressed proteins, such as Tom70, an outer mitochondrial membrane protein, or Sec7, a Golgi apparatus localized protein, 2E2-Envy expression from the stronger *RPL15A* promoter was critical for enhanced fluorescent imaging. On the other hand, the less abundant Pex3 protein, a peroxisomal membrane protein, required low expression of the 2E2-FP from the *URA3* promoter, as stronger 2E2-FP expression led to non-specific labeling.^1^ Furthermore, utilization of the weakly-expressed 2E2-(NLS)-FP versions are preferred during the labeling of nuclear proteins, as it can significantly improve the signal-to-noise ratio and thus detection efficiency.

### Problem 2

ConA does not dissolve completely. Even upon hours of mixing, the ConA solution is cloudy. When this happens, it can impede the subsequent filtering step.

### Potential solution

Avoid vortexing or heating the solution, but instead make sure that the pH of the ConA buffer is adjusted correctly (See materials and equipment). Alternatively, try ConA from a different provider.

### Problem 3

No colonies on agar plate after yeast transformation.

### Potential solution

Several factors can contribute to a yeast transformation failure. Initially, make sure that the plasmid containing 2E2-FP construct was successfully digested. Second, use a sufficient amount of digested 2E2-FP plasmid in the yeast transformation (at least 1 μg). During the day of the transformation, make sure cells will have at least 3 h to recover from lag phase, while never surpassing O.D._600_ = 0.6 upon dilution. Third, do not incubate cells for more than 1 h at 42°C during transformation, as this can lead to excessive heat shock and cell death. Fourth, do not skip or shorten the duration of the recovery step and always use prewarmed YPD medium. Finally, plate a sufficient amount of transformed cells on the selective plates, in several dilutions.

### Problem 4

Validation PCR failed.

### Potential solution

The most frequent reason for a failed validation PCR, apart from a negative colony or experimental mistake, is the dilution of excessive amount of yeast cells in the 20 mM NaOH solution. The solution should be slightly cloudy with yeast cells. Use the edge of a 100–200 μL tip to take a small amount of yeast from the plate. Additionally, primer design and amplification protocol should be performed according to manufacturer’s guidelines.

### Problem 5

Inaccurate identification and quantification of rings and/or foci.

### Potential solution

The parameters for rings and foci identification and quantification need to be carefully selected to achieve optimal fluorescence microscopy image analysis. First, the minimum and maximum values for rings “radii” must be set accordingly to ensure that all fluorescently-labeled ring structures in the imaging field will be accurately identified. Furthermore, the “sensitivity” parameter needs careful adjustment to avoid analysis artefacts (Figure 4C). Analogously, proper foci identification considerably depends on the chosen “noise” parameter value. If this value is very low, then a high amount of false positive foci will be detected (Figure 5E). On the other hand, if the “noise” value is very high, then none or only a few foci will be identified (false negative). Additionally, accurate foci intensity quantification lies in the appropriate selection of the parameters “pixels number” and the “radius” around the local maxima (strongest pixel within a region) that these pixels will be identified. Ideally, only pixels with significantly higher intensity relative to the fluorescent background around the foci should be taken into account.

## Resource availability

### Lead contact

Further information and requests for resources and reagents should be directed to and will be fulfilled by the lead contact, Amir Aharoni (aaharoni@bgu.ac.il).

### Materials availability

Plasmids for 2E2-FP integration and expression or yeast strains expressing 2E2-FP constructs, as well as plasmids for Envy, mKate2, Electra1, HALOtag, and -3HA or -6HA protein labeling generated in this study are available upon request. SiR-HALO dye samples are available upon request.

## Data Availability

• Microscopy images have been deposited at Zenodo and are publicly available as of the date of publication. DOIs are listed in the key resources table. Microscopy and any additional data reported in this paper will be shared by the lead contact upon request. • All original code has been deposited at Zenodo and is publicly available as of the date of publication. DOIs are listed in the key resources table. • Any additional information required to reanalyze the data reported in this paper is available from the lead contact upon request.
